# Therapeutic Stroke - an Interesting Case of Ischemic Stroke in Bilateral Thalamic and Subthalamic Regions Resulting in Reduction of Essential Tremor

**DOI:** 10.7759/cureus.1777

**Published:** 2017-10-16

**Authors:** Pradeep C Bollu, Chintan Rupareliya

**Affiliations:** 1 Department of Neurology, University of Missouri, Columbia, Missouri

**Keywords:** essential tremor, deep brain stimulation, ischemic stroke, thalamotomy, bilateral thalamic and subthalamic stroke

## Abstract

Essential tremor (ET) is the commonest adult-onset movement disorder, and its prevalence increases with age. About 10% of the patients with ET can be severely handicapped by it. Medical management is the first line of treatment for ET. In the past, refractory cases of ET underwent thalamotomy, a neurosurgical procedure that caused selective thalamic lesions. We describe a case of an elderly woman with ET that showed a dramatic improvement of her tremor after sustaining an acute stroke in bilateral thalamic and subthalamic regions. This ischemic insult essentially served the purpose of thalamotomy resulting in an improvement in her tremor.

## Introduction

Essential tremor (ET) is the commonest adult-onset movement disorder [1]. Its prevalence increases with age. About 10% of the patients with ET can be severely handicapped by it, and deep brain stimulation (DBS) has emerged as a treatment for patients with severe ET. The stereotactic target for DBS in treating ET is the ventral intermediate (ViM) nucleus of the thalamus. Prior to the advent of DBS, thalamotomy was the surgical method of choice for refractory ET but was ridden with multiple post-operative sequelae [2-3]. We describe a rare instance where the ischemic stroke that affected the thalamic and subthalamic regions bilaterally in a patient with ET resulted in a therapeutic effect on her tremor disorder.

## Case presentation

**Figure 1:**
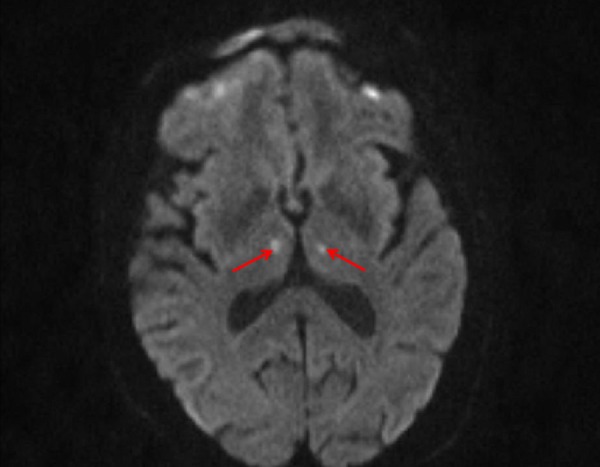
Magnetic resonance imaging (MRI) demonstrating bilateral medial thalamic infarcts Axial diffusion-weighted imaging (DWI) sequence showing restriction of diffusion in both medial thalami (red arrows)

**Figure 2:**
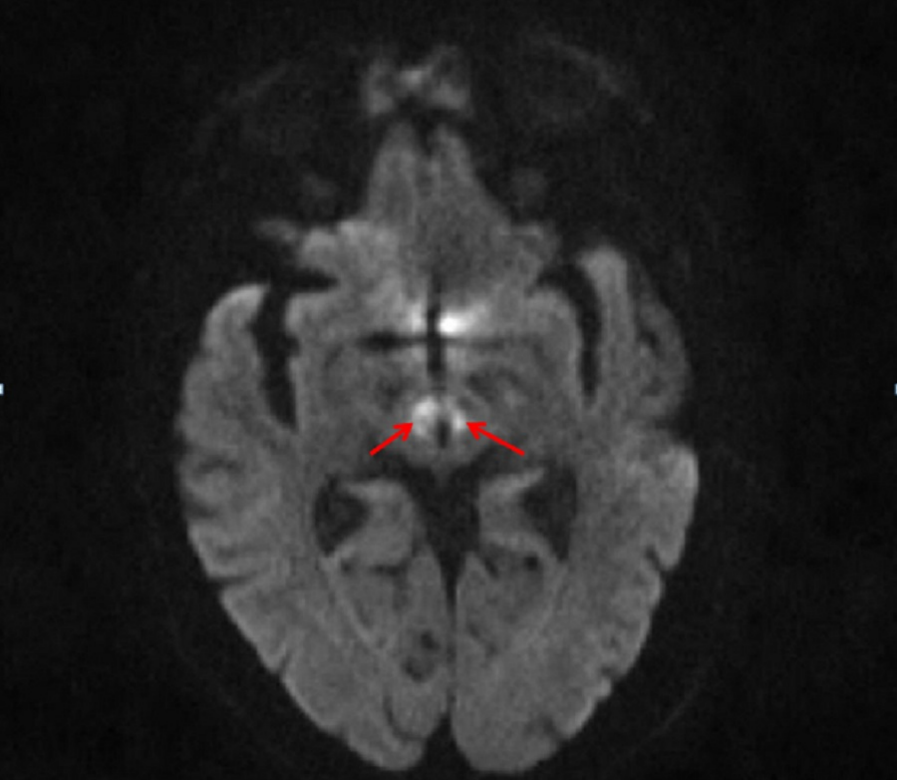
Magnetic resonance imaging (MRI) demonstrating stroke extension into subthalamic nucleus region Axial diffusion-weighted imaging (DWI) sequence showing extension of the stroke into the subthalamic region on both sides (red arrows)

An 87-year-old female patient presented to an outside hospital for acute onset of somnolence. Her past medical history was significant for hypertension and diabetes mellitus. Magnetic resonance imaging (MRI) of the brain performed at the outside facility showed the presence of bilateral thalamic (Figure 1) and subthalamic region (Figure 2) infarctions. Magnetic resonance angiography (MRA) of her head and neck did not reveal anomalous vasculature in the posterior circulation. The patient was drowsy for the first few days but improved gradually during the hospital stay. During the hospital course, both the patients and the relatives reported a marked reduction in her hand tremor for which she had been on propranolol. An institutional review board (IRB) approval from the University of Missouri was obtained for the publication of this case report. The IRB approval number for this case is 128125.

## Discussion

Essential tremor (ET) is the most common adult-onset movement disorder [1]. The tremor in this condition mainly affects upper extremities but can also affect lower extremities, head and occasionally, voice. A family history of similar tremor is seen in many cases which is why this condition is also called 'familial tremor' and 'familial essential tremor' [2]. In familial cases, it is inherited in an autosomal dominant fashion. The most common symptom is an action or postural hand or arm tremor that typically begins early in life. One of the important features of this condition is its significant response to alcohol consumption [3]. Most patients respond to medical treatments. Medications like Propranolol, Primidone, and Topiramate are used as the first line therapy, while benzodiazepines and gabapentin are used as second-line treatments [4]. Our patient also developed increasing sleepiness over the next few months which can be seen in the setting of bilateral thalamic insults [5]. 

In a small proportion of patients, the tremor is refractory to medical management and can significantly affect the patient's life. In the past, refractory cases of tremor were treated with thalamotomy [6]. However, this procedure had many longterm side effects including a significant effect on cognition [7]. With the advent of deep brain stimulation (DBS), thalamotomies are rarely performed. Traditionally, the ventralis intermedius nucleus of the thalamus is the stereotactic target for such surgery [4]. Our patient had bilateral thalamic and subthalamic infarctions which we propose simulated the thalamotomy. In her case, the MRA was unable to show any anomalous vasculature in the posterior circulation. Though the patient was unable to undergo a conventional angiogram, the simultaneous involvement of both the thalami and subthalamic regions suggests the possibility of the presence of a vascular anomaly, like the artery of Percheron [8].

## Conclusions

Our case is a very rare occurrence where an acute stroke actually helped with one of her other medical co-morbidities. The stroke in her thalamic and subthalamic nuclei on both sides mimicked the effect of thalamotomy, bringing on an improvement in her essential tremor. There are very few times where a stroke can be called a therapeutic stroke, and our case is one such rare instance where that is true.
